# *Cyclocarya paliurus* (Batal.) Ijinskaja Aqueous Extract (CPAE) Ameliorates Obesity by Improving Insulin Signaling in the Hypothalamus of a Metabolic Syndrome Rat Model

**DOI:** 10.1155/2017/4602153

**Published:** 2017-06-08

**Authors:** Guangyuan Xu, Hisae Yoshitomi, Wen Sun, Xuan Guo, Lili Wu, Xiangyu Guo, Lingling Qin, Yixin Fan, Tunhai Xu, Tonghua Liu, Ming Gao

**Affiliations:** ^1^Key Laboratory of Health Cultivation of the Ministry of Education, Beijing University of Chinese Medicine, Beijing 100029, China; ^2^School of Pharmaceutical Sciences, Mukogawa Women's University, 11-68 Koshien Kyuban-cho, Nishinomiya, Hyogo 663-8179, Japan; ^3^Department of Endocrinology, Dongfang Hospital, Beijing University of Chinese Medicine, Beijing 100078, China; ^4^School of Chinese Pharmacy, Beijing University of Chinese Medicine, Beijing 100029, China

## Abstract

**Background:**

Antiobesity drugs may not be optimal for treating obesity. However novel antiobesity agents, especially those derived from natural products, may be suitable. Therefore, we investigated the effects and mechanisms of* Cyclocarya paliurus* (CP) aqueous extract (CPAE) on obesity.

**Methods:**

SHR.Cg-Leprcp/NDmcr (SHR/cp) rats were used as a model of obesity and metabolic syndrome. Experimental animals were allocated into two groups—control and CPAE (0.5 g/kg)—for a 7-week treatment period. Examinations were performed, including general physiological characteristics, obesity-related biochemical parameters, and insulin-signaling pathway-related proteins in the hypothalamus.

**Results:**

Treatment with CPAE reduced food intake, body weight, organ weight, fat mass, and body mass index (BMI) in SHR/cp rats. Meanwhile, CPAE also decreased the levels of fasting serum glucose, fasting serum insulin, HOMA-IR, serum free fatty acids, serum malondialdehyde, serum superoxide dismutase, and serum total-glutathione. The levels of phosphorylation of target proteins—including InsR, IRS1, PI3Kp85, Akt, and FoXO1 as well as protein expression of POMC—were significantly upregulated in the hypothalamus, but NPY expression remarkably decreased.

**Conclusions:**

CPAE has antiobesity, antihypoglycemic, antihypolipidemic, and antioxidant properties. The mechanism responsible for the antiobesity effect of CPAE may be related to suppression of energy intake via regulation of insulin-signaling pathway in the hypothalamus.

## 1. Introduction

Obesity is a major public health problem approaching epidemic proportions worldwide. It is associated with metabolic comorbidities such as type 2 diabetes mellitus, cardiovascular injury, hyperlipemia, and other obesity-related diseases [1, 2]. Therefore, a better understanding of the mechanisms of obesity is urgently needed along with the development of new antiobesity drugs.

The imbalance between energy intake and energy expenditure is the key cause of obesity [3, 4]. Previous studies revealed that the hypothalamus is a key brain region in the regulation of food intake, and adipose tissue is crucial in energy storage and expenditure. Both play significant roles in maintaining energy homeostasis and in treating obesity [5–7].

In the hypothalamus, there are two types of appetite-regulating neurons: (a) coexpression of neuropeptide Y (NPY) and agouti-related protein (AgRP), both of which express melanocortin receptors (MCR) antagonists, and (b) proopiomelanocortin (POMC) which expresses MCR agonists [8, 9]. In the hypothalamus, POMC and NPY neurons are regulated by different hormones (e.g., leptin and insulin). These hormones make the melanocortin system sensitive to changes in both food intake and body weight [10]. Since insulin receptors (InsR) are widely expressed in the hypothalamus, the insulin-signaling pathway plays a key role in the control of feeding activity [11]. Two studies found that diabetes and stress are involved in the dysregulation of insulin-signaling pathway in the hypothalamus [12, 13]. Another study showed that intracerebroventricular (i.c.v.) injection of insulin can inhibit food intake through the regulation of PI3K signaling in the hypothalamus [14]. Moreover, in the hypothalamus, the forehead box-containing protein of the O subfamily (FoxO)1—a downstream transcription factor of the insulin-signaling pathway—has a dual function: inhibiting the expression of POMC and stimulating the expression of NPY leading to an increased food intake [15, 16]. On the other hand, FoxO1 activities are also regulated by the InsR/PI3K/Akt signaling pathway, in which insulin induces Akt phosphorylation, followed by inactivation of FoxO1 due to its phosphorylation by p-Akt [17].

Inactivation of FoxO1 causes upregulation of POMC expression and downregulation of NPY expression, leading to loss of appetite and weight [18]. On the other hand, insulin resistance in the hypothalamus reduces insulin-mediated Akt phosphorylation, thereby upregulating overexpression of FoxO1, which results in increased food intake and obesity [19, 20]. Therefore, to control food intake in obesity, targeting the insulin-signaling pathway may be a suitable approach.

Therapeutic intervention via weight-loss drugs (accompanied by diet and exercise) is one option for the treatment and management of obesity. However, antiobesity drugs (e.g., orlistat) currently available or under development in clinical trials may not be optimal for treating morbid obesity with metabolic complications. Thus it may be beneficial to develop novel antiobesity agents, especially those derived from natural products, which often have fewer adverse effects.

*Cyclocarya paliurus* (CP) (Batal.) Iljinsk (family Cyclocaryaceae) is a traditional medicinal herb, which only grows in southern China [21, 22]. The leaves of CP (commonly known as “sweet tea tree”) have been widely used in traditional medicine for the treatment of hypertension, obesity, and diabetes. In addition, CP hypoglycemic tea is the first Chinese health tea approved by the United States Food and Drug Administration (FDA) [23, 24].

CP aqueous extract (CPAE), one of the main active components of CP, contains many polysaccharide components. Several studies showed that CPAE can improve insulin resistance by inhibiting serine phosphorylation of IRS-1, thereby restoring tyrosine phosphorylation of IRS-1 and increasing Akt phosphorylation in the muscle tissue of mice with adipose dysfunction and insulin resistance [25]. In addition, animal studies showed that CPAE can reduce food intake in high-fat-diet (HFD) and streptozotocin- (STZ-) induced type 2 diabetic rats and can lower lipid levels in hyperlipidemic mice [26, 27]. However, it is not clear whether CPAE can affect insulin signaling in the hypothalamus. Moreover, the mechanisms underlying the antiobesity effects of CPAE are also rarely reported. Therefore, by identifying the responsible mechanism and possible effect of CPAE on food intake, we can elucidate our knowledge of obesity treatment using CPAE and measure its efficacy.

In this study, we investigated the mechanism and effect of CPAE on obesity using leptin receptor-deficient SHR.Cg-Leprcp/NDmcr (SHR/cp) rats as a model of morbid obesity with metabolic syndrome (MetS) [28]. Analysis of our results would then reveal the effect of CPAE on the insulin-signaling pathway in the hypothalamus of the leptin receptor-deficient rats.

## 2. Materials and Methods

### 2.1. Preparation of CPAE

CP leaves were collected from Xiushui County, Jiangxi Provence, and verified at the School of Chinese Materia Medica of Beijing University of Chinese Medicine (Beijing, China) by Professor Tun-hai Xu. The air-dried and powdered leaves (40 kg) were extracted 3 times (2 hours/each) with 8 times the amount of 85% ethanol-heating reflux. The extracts were combined, concentrated, and finally dissolved in 40 L water, followed by a degreasing treatment (flushed 3 times with petroleum ether at a 1 : 1 volume ratio). The water extracts were then freeze-dried after decompression and concentration, leaving the CPAE powder (600 g) [29, 30]. The proportion of total polysaccharides in the CPAE powder was approximately 68.11%, as determined by UV-spectrometer.

### 2.2. Animal Experiment

Male 7-week-old SHR/cp rats (190–210 g) and age-matched male Wistar Kyoto (WKY) rats (150–170 g) were purchased from Japan SLC, Inc. (Shizuoka, Japan). The animals were kept in a specific-pathogen-free (SPF) animal facility at Mukogawa Women's University (Nishinomiya, Japan), given ad libitum access to normal chow and water, and maintained in controlled temperature (22–24°C), relative humidity (40–60%), and 12/12 h light/dark cycle conditions. The care and treatment of the experimental animals were conducted in accordance with the Guidelines for the Care and Use of Laboratory Animals of Mukogawa Women's University. The experimental protocols were approved by the Animal Care and Use Committee of Mukogawa Women's University.

SHR/cp rats were a proven model of prediabetes and MetS characterized by hyperglycemia, hyperlipidemia, hypertension, and obesity [26]. The SHR/cp rats were chosen (see Table 1) and randomly divided into two groups, each containing 6 rats: (1) an untreated control group (control group) and (2) rats treated with 0.5 g/kg CPAE (CPAE group). Levels of fasting blood glucose (FBG), total triglycerides (TG), total cholesterol (TC), and free fatty acids (FFA) in the serum, as well as animal body weight, were measured in all rats. CPAE was prepared in sterile water and administered orally once per day for 7 consecutive weeks. Control group rats received the same volume of sterile water.

### 2.3. General Condition, Organ Weight, and Body Mass Index (BMI) of Experimental Rats

The general condition of the experimental rats was daily monitored, including mental state, body weight, body weight gain, food intake, and survival. Body weight and food intake were recorded every day throughout the experiments. Organs and fat of experimental rats were weighed at the end of treatment. BMI was measured by bioelectrical impedance analysis (BIA) (ImpediVet, ImpediMed Ltd., Brisbane, Australia). Acquired data were processed using bioimpedance software (ImpediVet Vet BIS1 v1.0.2).

### 2.4. Biochemical Analysis

TG, TC, FFA, glutamic pyruvic transaminase (GPT), and glutamic oxaloacetic transaminase (GOT) were measured using commercially available kits (Wako Pure Chemical Industries, Osaka, Japan). Serum creatinine (Screa), malondialdehyde (MDA), superoxide dismutase (SOD), and total-glutathione (T-GSH) were determined by colorimetric assays (SINO-UK Institute of Biological Technology, Beijing, China). The level of serum insulin was analyzed using enzyme-linked immunosorbent assay (ELISA) kits (SINO-UK Institute of Biological Technology, Beijing, China). Quantification of insulin resistance and beta-cell function was performed using the homeostasis model of assessment-insulin resistance (HOMA-IR) index calculated from the equation: HOMA-IR = FPG × Fins/22.5 [31].

### 2.5. Histological Examination of Adipose Tissue

Abdominal adipose tissues were isolated at the end of treatment and fixed in 4% paraformaldehyde. Tissue samples were embedded in paraffin, sectioned (4 *μ*m thick), stained with hematoxylin-eosin (H&E), and examined under an optical microscope (Olympus BX53, Olympus, Tokyo, Japan).

### 2.6. Western Blot Analysis

Hypothalamus and adipose tissue samples were collected at the end of treatment and stored at −80°C prior to use. Tissue samples were prepared in a homogenization buffer containing 50 mM Tris–HCl (pH 7.4), 100 mM NaCl, 1% Nonidet-P40, 0.25% sodium deoxycholate, 0.1% SDS, 1 mM EDTA, 50 mM NaF, 2 mM Na_3_VO_4_, 30 mM sodium pyrophosphate, 2 mM PMSF, 1 mM benzamidine, 0.02 g/mL trypsin inhibitor, 0.02 g/mL leupeptin, and 0.02 g/mL aprotinin. Lysates were incubated on ice for 30 min, followed by centrifugation at 15,000 rpm for 30 min to collect the supernatant. After measurement of protein concentration, supernatants with equal amount of protein were mixed with a loading buffer containing 0.5 mmol/L Tris/HCl, pH 6.8, glycerol, 10% SDS, 0.1% bromophenol blue, and 2-mercaptethanol and then denatured in a boiling water bath.

The protein samples were separated by 7.5%–12.5% SDS-PAGE gel electrophoresis, transferred onto a PVDF membrane (Amersham Life Science Inc., Little Chalfont, Buckinghamshire, UK). After being blocked using either Blocking One or Blocking One-p buffer (Nacalai Tesque, Kyoto, Japan), the membranes were incubated at 4°C overnight with the primary antibodies, including p-InsR (cat#, 3024s), PI3Kp85 (4257s), p-Akt (4060s), p-FoXO1(9461s), and *β*-actin antibodies (4970s) (Cell Signaling Technology, USA), as well as POMC (ab94446), NPY (ab180809) (Abcam, Eugene, OR, USA), and p-IRS1tyr989 (L1912) (Santa Cruz Biotechnology Inc., Dallas, TX, USA). The next day, membranes were washed with TTBS and incubated for 1 hour with HRP conjugated anti-rabbit, anti-goat, or anti-mouse IgG antibodies (1 : 10,000 dilution). The bound antibodies were visualized using enhanced chemiluminescence. The relative levels of interesting proteins to *β*-actin (internal control) were analyzed by ImageJ v7.0 software.

### 2.7. Statistical Analysis

Data are expressed as mean ± standard deviation (SD). The difference between two groups was analyzed by Student's* t*-test. For multiple comparisons, the significance of differences among groups was determined by one-way analysis of variance with Bonferroni's correction. *P* < 0.05 was considered statistically significant.

## 3. Results

### 3.1. Effects of CPAE on Obesity and MetS in SHR/cp Rats

**Figure 1:**
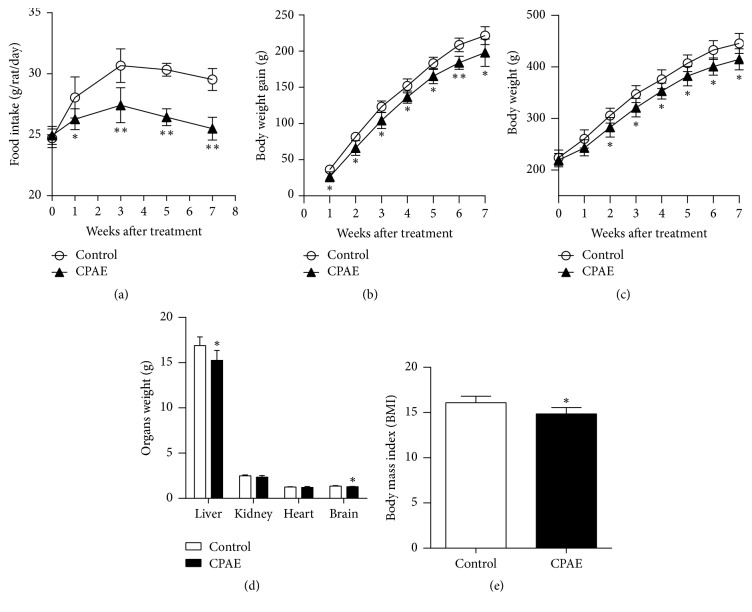
*CPAE ameliorates obesity by control of appetite in SHR/cp rats*. (a) Body weight, (b) body weight gain, (c) food intake (g/rat/day), (d) organ weight, and (e) body mass index (BMI) of SHR/cp rats treated with 0.5 g/kg CPAE or untreated controls (*n* = 6). Values are expressed as the mean ± SD. ^*∗*^*P* < 0.05 and ^*∗∗*^*P* < 0.01 versus control group. *n* = 6 per group.

As shown in Figure 1, after 1 week of treatment, food intake in CPAE treatment group was significantly lower than that in control group (Figure 1(a)). Therefore, body weight gain in the CPAE group was smaller than that in control group after 1 week of treatment (Figure 1(b)). Moreover, an increase in body weight was found in both groups, but the amount of increased body weight in CPAE group was significantly smaller than that in control group after 2 weeks' treatment (Figure 1(c)). Comparisons of the organ weight differences between the two groups showed that the liver and brain weights in the CPAE group were significantly lower than in the CPAE group after 7 weeks' intervention (Figure 1(d)). BMI in the CPAE group was also significantly lower than in control group rats (Figure 1(e)).

**Figure 2:**
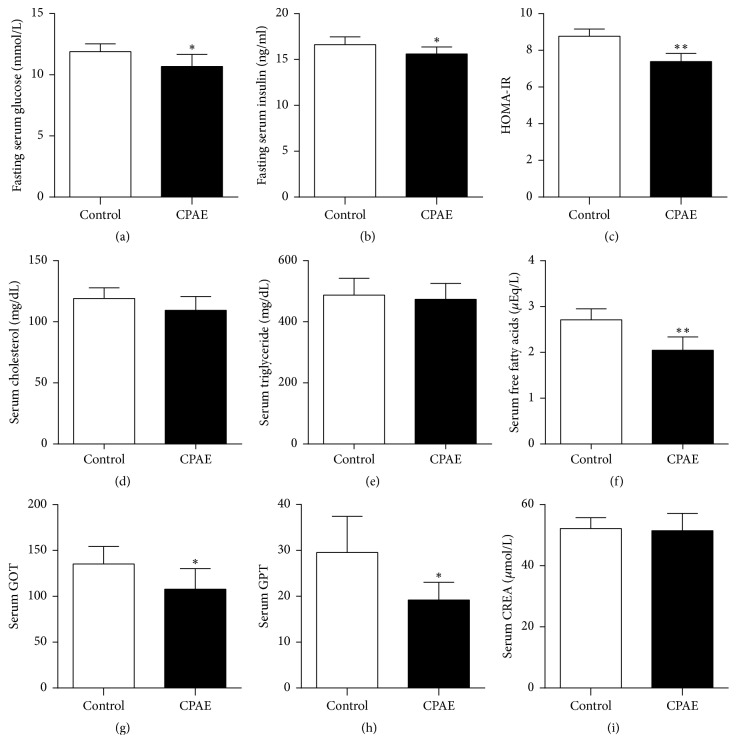
*CPAE treatment affects glucose and lipid levels and hepatorenal function in SHR/cp rats*. (a) Fasting serum glucose, (b) fasting serum insulin, (c) HOMA-IR, (d) serum cholesterol, (e) serum triglyceride, (f) serum free fatty acids, (g) serum GOT, (h) serum GPT, and (i) serum CREA of SHR/cp rats treated with 0.5 g/kg CPAE or untreated controls (*n* = 6) were measured as described in Materials and Methods. Values are expressed as the mean ± SD. ^*∗*^*P* < 0.05 and ^*∗∗*^*P* < 0.01 versus control group. *n* = 6 per group.

As shown in Figure 2, compared to those in the control group, the fasting serum glucose, fasting serum insulin, and HOMA-IR in the CPAE group were all significantly decreased after 7 weeks' intervention (Figures 2(a), 2(b), and 2(c)). The levels of serum cholesterol, triglyceride, and free fatty acids in the CPAE group were slightly lower than those in control group; only the difference of serum free fatty acids between two groups was statistically significant (Figures 2(d), 2(e), and 2(f)). In addition, the levels of serum GPT and GOT were significantly reduced in the CPAE group, but the level of serum creatinine was not statistically different between two groups (Figures 2(g), 2(h), and 2(i)), thus indicating CPAE has no hepatorenal toxicity. Taken together, these data revealed that CPAE can improve obesity, insulin resistance, hyperlipoidemia, and hyperglycemia in SHR/cp rats.

### 3.2. Antioxidant Potential of CPAE in SHR/cp Rats

**Figure 3:**
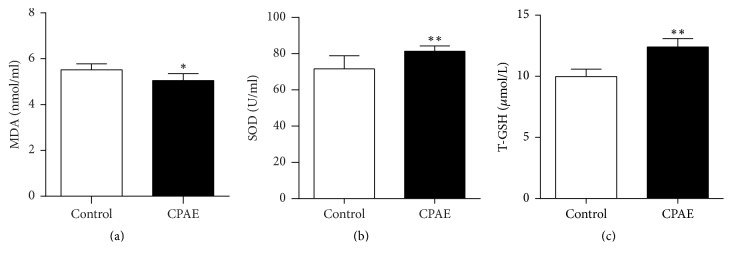
*Antioxidant potential of CPAE in SHR/cp rats*. (a) MDA, (b) SOD, and (c) the level of T-GSH of SHR/cp rats treated with 0.5 g/kg CPAE or untreated controls (*n* = 6) were measured as described in Materials and Methods. Values are expressed as the mean ± SD. ^*∗*^*P* < 0.05 and ^*∗∗*^*P* < 0.01 versus control group. *n* = 6 per group.

The antioxidant potential of CPAE in SHR/cp rats is shown in Figure 3. After treatment with CPAE for 7 weeks, the MDA level in the CPAE group was significantly lower than that in control group (Figure 3(a)). Meanwhile, the levels of SOD and T-GSH in CPAE group were significantly higher than those in the control group (Figures 3(b) and 3(c)). Those results demonstrated the antioxidant potential of CPAE in SHR/cp rats.

### 3.3. Effects of CPAE on Fat Mass in SHR/cp Rats

**Figure 4:**
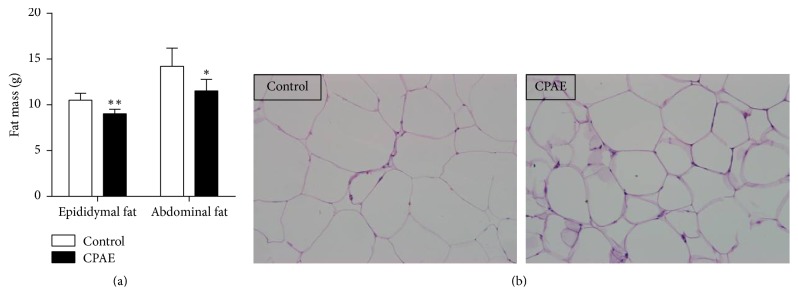
*CPAE reduces fat mass in SHR/cp rats*. (a) Measurement of fat mass and (b) representative H&E staining results (magnification ×200) of the adipose tissue collected from SHR/cp rats which were treated with 0.5 g/kg CPAE or untreated controls. Values are expressed as the mean ± SD. ^*∗*^*P* < 0.05 and ^*∗∗*^*P* < 0.01 versus control group. *n* = 6 per group.

After 7 weeks' treatment with CPAE, the fat mass of either the epididymal adipose tissue or the abdominal adipose tissue was significantly reduced, compared to the control group (Figure 4(a)). Furthermore, cell size of abdominal adipocyte in CPAE group was also decreased, compared to the control group (Figure 4(b)). These results could be attributed to the inhibitory effects of CPAE on appetite.

### 3.4. Effects of CPAE on the Insulin-Signaling Pathway in the Hypothalamus of SHR/cp Rats

**Figure 5:**
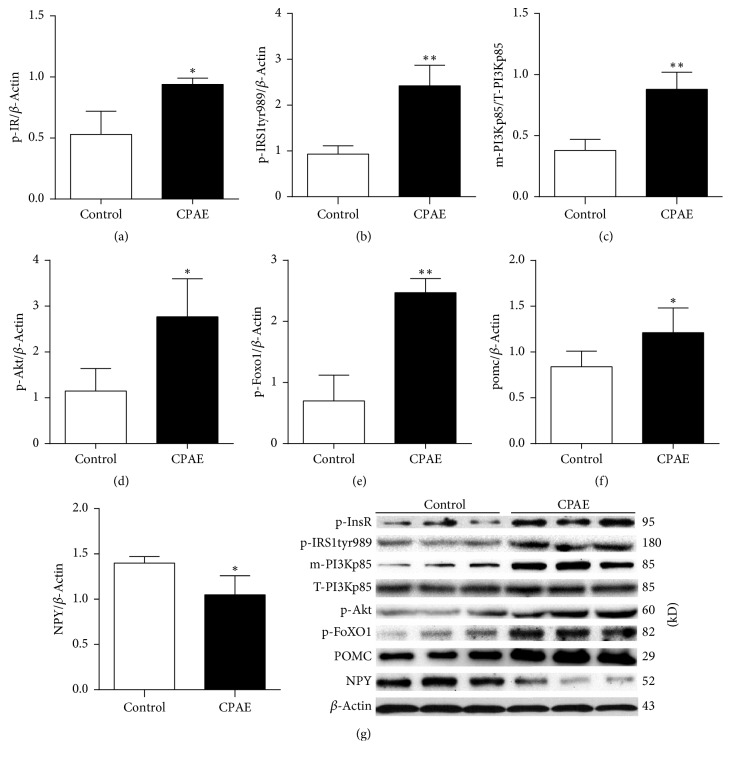
*CPAE activates the insulin-signaling pathway in the hypothalamus of SHR/cp rats*. (a) The phosphorylation level of InsR, (b) the phosphorylation level of IRS1tyr989, (c) the level of m-PI3Kp85, (d) the phosphorylation level of Akt, (e) the phosphorylation level of FoXO1, (f) the protein expression level of POMC, and (g) NPY of SHR/cp rats treated with 0.5 g/kg CPAE or untreated controls. All of the results were determined by Western blot analysis as described in Materials and Methods. Values are expressed as the mean ± SD. ^*∗*^*P* < 0.05 and ^*∗∗*^*P* < 0.01 versus control group. *n* = 6 per group.

Insulin signaling plays an important role in the regulation of gene expression of POMC and NPY. Moreover, InsR and IRS1 are key molecules in the insulin-signaling pathway involved in the activation of downstream targets. To investigate the effects of CPAE on the insulin-signaling pathway in the hypothalamus, activities of several important molecules in this pathway were examined by Western blot analysis. Results showed that phosphorylation levels of InsR and IRS1 (tyr989) in CPAE group were markedly increased, compared to the control group (Figures 5(a) and 5(b)). PI3K, which is activated by upstream p-IRS1 (tyr989), upregulates its downstream target (Akt phosphorylation). PI3K exhibits its effects by translocating to the cell membrane from the cytoplasm [32, 33]. Our study found that the level of cell membrane PI3Kp85 (m-PI3Kp85) in the CPAE group was significantly higher than in the control group (Figure 5(c)). Thus, the level of p-Akt in CPAE group was higher than that in the control group (Figure 5(d)).

FoxO1 is a transcription factor that is abundant in two neuronal subpopulations characterized by expression of POMC or NPY [34]. FoxO1 is inactivated by Akt, whereas activation of FoxO1 promotes food intake by reducing POMC expression and inducing NPY expression. Activation of the insulin-signaling pathway results in the upregulation of p-Akt and sequentially inactivation of FoxO1 due to its phosphorylation by Akt, leading to loss of appetite related to upregulated POMC and downregulated NPY [18].

Our results showed that p-FoxO1 was significantly increased in the CPAE group, compared to the control group. Therefore, POMC expression in the CPAE group was significantly higher than in the control group (Figure 5(f)), although the expression of NPY was decreased (Figure 5(g)). These results demonstrated that CPAE treatment can activate the insulin-signaling pathway in SHR/cp rats.

## 4. Discussion

Obesity is a chronic endocrine metabolic disease associated with hyperglycemia, hyperlipemia, and hypertension [35]. Physically, excessive energy intake due to high food intake is the main cause of obesity. On the other hand, the hypothalamus, a principal center in the brain for regulating appetite, plays an important role in regulation of energy intake [36]. In addition, the insulin-signaling pathway in the hypothalamus is involved in the regulation of appetite [37].

It has been reported that the CPAE can improve insulin resistance by affecting the insulin-signaling pathway in peripheral tissue. However, it is not clear whether CPAE also regulates the insulin-signaling pathway in the hypothalamus. Therefore, we investigated the effect of CPAE on the insulin-signaling pathway in hypothalamus, using SHR.Cg-Leprcp/NDmcr (SHR/cp) rats as a model of obesity and MetS.

In the present study, we demonstrated that treatment with CPAE (0.5 g/kg daily for 7 weeks) can improve obesity, diabetes, hyperglycemia, and hyperlipemia in SHR/cp rats. Our results suggest the effects of CPAE on obesity may be dependent on its ability to decrease food intake. Therefore, a better understanding of the mechanism of antiobesity effects of CPAE is helpful in developing novel antiobesity drugs for treatment of morbid obesity.

The levels of POMC and NPY expression in the hypothalamus are closely associated with the control of food intake. Moreover, the expressions of both POMC and NPY are also controlled by the insulin-signaling pathway in hypothalamus, because NPY neurons are insulin-responsive and appetite-stimulating. Indeed, NYP expression can be inhibited by insulin. POMC decreases food intake and reduces body weight in response to insulin stimulation, but the function of POMC is inhibited by neighboring NPY neurons [38, 39]. Previous studies found that insulin receptor (InsR) is widely expressed in the hypothalamus and is involved in the regulation of the insulin-stimulated downstream signaling, while the PI3K/Akt/FoxO1 axis is a key downstream-signaling pathway of InsR and is related to the control of appetite [14].

It is well known that PI3K-dependent Akt activation inhibits FoxO1 activity. Insulin binds to receptors and increases phosphorylation of Akt (one of its downstream targets). Inactivation of FoXO1 by Akt then follows, resulting in upregulation of POMC and downregulation of NPY expression, leading to loss of appetite [19, 40–42]. Therefore, in this study we paid special attention to relationship between CPAE and expression of POMC and NPY. Our data revealed that after 7 weeks' CPAE treatment the level of POMC expression was increased, but NPY expression was reduced. Furthermore, key molecules in the insulin-signaling pathway such as InsR, PI3K, Akt, and FoxO1 were activated or inactivated through their phosphorylation. In our study, changes in the phosphorylation of those molecules were found in CPAE-treated SHR/cp rats. In the experimental rats, those CPAE-mediated changes were correlated with decreased food intake and reduced body weight.

In summary, in this animal study we have demonstrated that CPAE can reduce obesity and insulin resistance and also reduce hyperglycemic, hyperlipidemic, and oxidant stress. Those results are consistent with previously reported studies. We further found that the antiobesity function of CPAE is associated with the suppression of excessive energy intake through a mechanism by activating the insulin-signaling pathway, leading to the inactivation of FoXO1, followed by the upregulation of POMC expression and the downregulation of NPY expression.

Our findings suggest that CPAE may prove to be a promising agent for obesity treatment through regulation of the feeding center of the hypothalamus. In the future, we will further investigate active ingredients and properties of CPAE.

## Figures and Tables

**Table 1:** SHR-cp rats used in the experiments.

Group	Dose (g/kg)	FBG (mg/dL)	Body weight (g)	TG (mg/mL)	CHO (mg/dL)	FFA (mEq/L)	SBP (mmHg)
Control	—	94.57 ± 10.23	172.17 ± 8.50	274.13 ± 68.08	64.60 ± 7.15	1.96 ± 0.16	130.78 ± 10.57
CPAE	0.5	92.10 ± 9.50	171.50 ± 5.09	275.66 ± 48.87	64.08 ± 6.91	1.96 ± 0.28	131.28 ± 5.37

Data are expressed as the mean ± SD.

*P* < 0.05 and ^*∗∗*^*P* < 0.001 versus control. Control, control group (*n* = 6); CPAE, *Cyclocarya paliurus* leaf aqueous extract group (*n* = 6); SHR/cp, SHR.Cg-Leprcp/NDmcr rat. FBG, fasting blood glucose; TG, total triglycerides; CHO, total cholesterol; FFA, free fatty acids; SBP, systolic blood pressure.
